# Tilianin Reduces Apoptosis *via* the ERK/EGR1/BCL2L1 Pathway in Ischemia/Reperfusion-Induced Acute Kidney Injury Mice

**DOI:** 10.3389/fphar.2022.862584

**Published:** 2022-06-03

**Authors:** Zengying Liu, Chen Guan, Chenyu Li, Ningxin Zhang, Chengyu Yang, Lingyu Xu, Bin Zhou, Long Zhao, Hong Luan, Xiaofei Man, Yan Xu

**Affiliations:** ^1^ Department of Nephrology, the Affiliated Hospital of Qingdao University, Qingdao, China; ^2^ Medizinische Klinik und Poliklinik IV, Klinikum der Universität, LMU München, München, Germany

**Keywords:** tilianin, acute kidney injury, ischemia–reperfusion injury, ERK, EGR1, apoptosis

## Abstract

**Background:** Acute kidney injury (AKI) is a common syndrome impacting about 13.3 million patients per year. Tilianin has been reported to alleviate myocardial ischemia/reperfusion (I/R) injury, while its effect on AKI is unknown; thus, this study aimed to explore if tilianin protects I/R-induced AKI and the underlying mechanisms.

**Methods:** The microarray dataset GSE52004 was downloaded from GEO DataSets (Gene Expression Omnibus). Differential expression analysis and gene-set enrichment analysis (GSEA) were performed by R software to identify apoptosis pathway-related genes. Then, *RcisTarget* was applied to identify the transcription factor (TF) related to apoptosis. The STRING database was used to construct a protein–protein interaction (PPI) network. Cytoscape software visualized PPI networks, and hub TFs were selected via *cytoHubba.* AutoDock was used for molecular docking of tilianin and hub gene-encoded proteins. The expression levels of hub genes were assayed and visualized by quantitative real-time PCR, Western blotting, and immunohistochemistry by establishing I/R-induced AKI mouse models.

**Results:** Bioinformatics analysis showed that 34 genes, including FOS, ATF4, and Gadd45g, were involved in the apoptosis pathway. In total, seven hub TFs might play important roles in tilianin-regulating apoptosis pathways. In *in vivo*, tilianin improved kidney function and reduced the number of TUNEL-positive renal tubular epithelial cells (RTECs) after I/R-induced AKI. Tilianin reduced the activation of the ERK pathway and then downregulated the expression of EGR1. This further ameliorated the expression of anti-apoptotic genes such as BCL2L1 and BCL2, reduced pro-apoptotic genes such as BAD, BAX, and caspase-3, and reduced the release of cytochrome c.

**Conclusion:** Tilianin reduced apoptosis after I/R-induced AKI by the ERK/EGR1/BCL2L1 pathway. Our findings provided novel insights for the first time into the protective effect and underlying molecular mechanisms of tilianin on I/R-induced AKI.

## Introduction

Acute kidney injury (AKI), a common clinical syndrome, is defined as a rapid decrease in kidney function (Kellum et al., 2013; Yu and Feng, 2021; Zhong and He, 2021), with 10–15% of inpatients suffering from it, while the frequency is up to 50% in the intensive care unit (Al-Jaghbeer et al., 2018). As one of the major etiology of AKI, ischemia/reperfusion (I/R)-induced AKI develops in the context of many clinical conditions such as kidney transplantation, cardiac, and vascular surgery (Lameire et al., 2013). Patients with AKI are at an increased risk of chronic kidney disease (Mantovani and Zusi, 2020; Nikolic-Paterson et al., 2021; Xiong et al., 2021), and repeated episodes of AKI contribute to chronic kidney disease progression. Despite great progress in the understanding of the etiologies and pathological mechanisms of AKI, it remains an unmet medical need and affects 13.3 million patients annually worldwide (Mehta et al., 2015; Zuk and Bonventre, 2016).

Renal tubular epithelial cells (RTECs) are the most abundant cells in the kidney, which are densely packed with mitochondria where adenosine triphosphate (ATP) is generated (Tammaro et al., 2020; Li et al., 2021). Once the kidney is suffering from hypoxia and reoxygenation, ATP depletion with the reactive oxygen species (ROS) leads to the RTEC injury and then apoptosis (Ishimoto and Inagi, 2016; Wei et al., 2020), which results in kidney failure. Apoptosis, the key pathophysiological process in I/R-induced AKI (Linkermann et al., 2014), is triggered by either the extrinsic or the intrinsic (mitochondrial or BCL2-regulated) pathways (Bedoui et al., 2020). The imbalance of pro-apoptotic and pro-survival members of the BCL2 protein family leads to mitochondrial outer-membrane permeabilization (MOMP), which allows the consequent release of cytochrome c (Cyt C) and activates caspases (Bedoui et al., 2020; Bock and Tait, 2020). Furthermore, evidence has confirmed the implication of the Ras/Raf/ERK pathway in the induction of apoptosis, but the role of this pathway in I/R-induced AKI is still unclear.

Mounting evidence has suggested that compounds isolated and identified from natural products were considered sources of new drugs (Wang et al., 2018; Howes et al., 2020; Izzo et al., 2020; Miao et al., 2020; Newman and Cragg, 2020; Qin et al., 2020; Zubcevic, 2020; Wang et al., 2021). Tilianin (acacetin-7-glucoside), a bioactive flavonoid glycoside compound isolated from various medicinal plants, such as *Agastache rugosa* (Akanda et al., 2019), has a wide range of pharmacological and biological activities of inhibition of inflammation and cell apoptosis (Akanda et al., 2019; Tian et al., 2019). Studies showed that tilianin alleviates myocardial I/R-induced injury through mitochondria protection and inhibition of apoptosis (Wang et al., 2017; Zeng et al., 2018). However, whether tilianin protects kidney function of I/R-induced AKI mice is elusive. Therefore, this study aimed to 1) explore the possible protective effects on I/R-induced renal dysfunction, 2) speculate the underlying mechanisms of anti-apoptosis using bioinformatics methods, and 3) validate the predicted mechanisms using mice as an experimental model.

## Methods and Materials

### Tilianin

**FIGURE 1:**
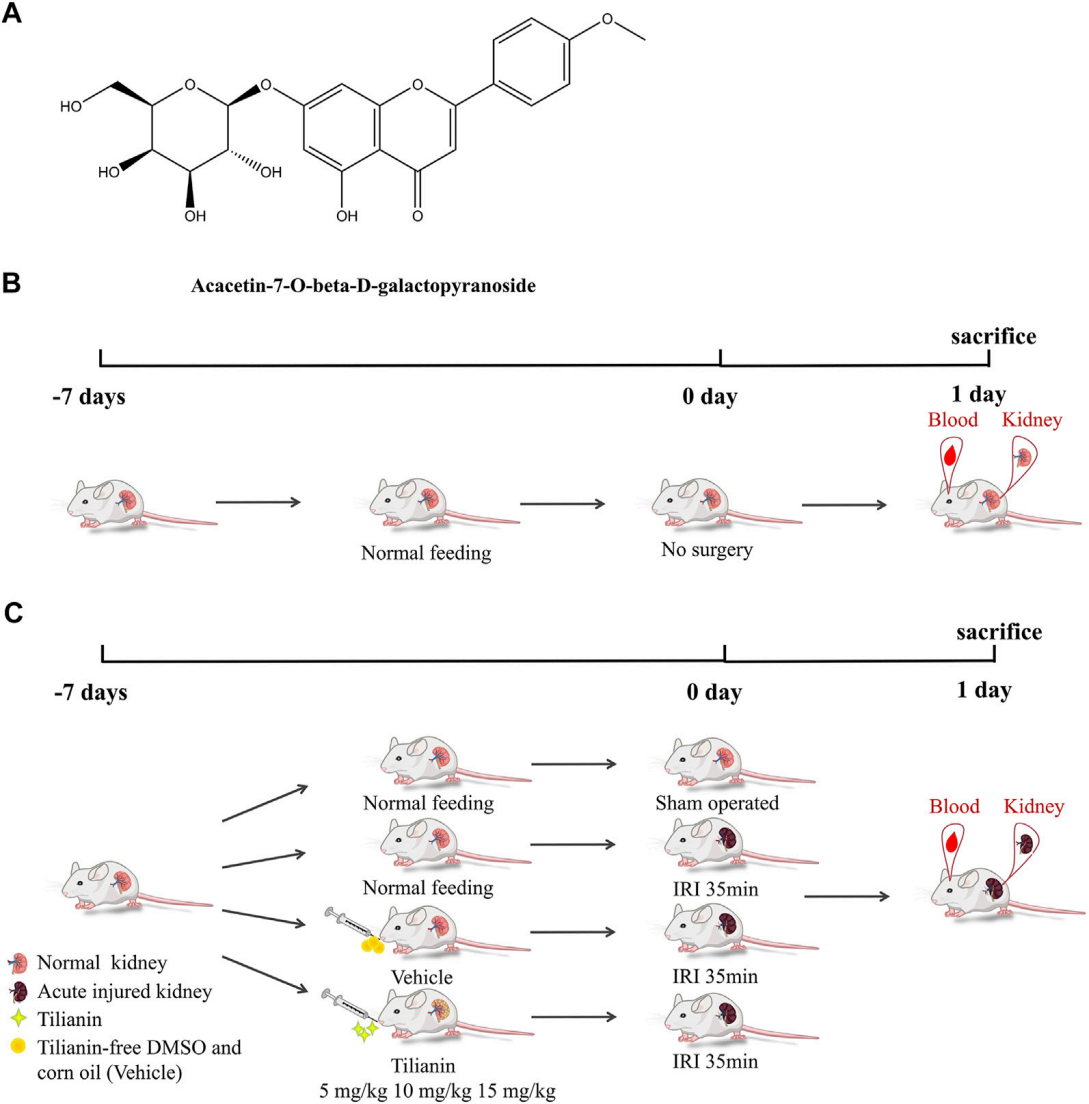
Tilianin and schematic diagram of I/R-induced AKI model establishment, treatment processes, and therapy assessments. **(A)** Chemical structure of acacetin-7-O-beta-d-galactopyranoside (tilianin). **(B)** Mice in the control group were fed normally and experienced no renal vascular pedicles. **(C)** Mice in the sham group were subjected to laparotomy and did not suffer from AKI. Groups of I/R-induced AKI mice were fed normally or tilianin (i.g., 5 mg/kg, 10 mg/kg, and 15 mg/kg, once a day, the total for 7 days) or vehicle (i.g., 10 ml/kg, once a day, the total for 7 days).

Tilianin (Figure 1A) was purchased from MCE company (CAS: 4291-60-5, cat: HY-N2555, purity: 99.57%, MedChemExpress, NJ, United States). The molecular formula of tilianin is C₂₂H₂₂O₁₀, and the molecular weight is 446.4. Tilianin was dissolved in dimethyl sulfoxide (DMSO, cat: D8370, Solarbio, Shanghai, China) with the concentration of stock liquid at 15 mg/ml, and then corn oil was used to dilute the stock liquid in *in vivo* experiments.

### Animals and Treatment

Male C57BL/6 mice were purchased from Jinan Pengyue Experimental Animal Breeding Co., Ltd., (Jinan, China), aged 6–8 weeks, and weighing 18–20 g. All mice were housed in a temperature-controlled room (23 ± 2°C) with 50% humidity and a 12-h light/dark cycle. The mice were divided into seven groups (three per group), including a control group, a sham operation group, an ischemia–reperfusion injury (IRI) group, three IRI groups treated with three doses of tilianin: 5 mg/kg, 10 mg/kg, and 15 mg/kg, and an IRI group treated with tilianin-free DMSO and corn oil. The mice were given intragastric administration for 7 days before surgery, at a volume of 10 ml/kg weight once a day. Surgery was performed 1 h after the last administration. The IRI model was established by clamping the bilateral renal vascular for 35 min using microvascular clamps (Roboz) and then reperfusion. The sham operation group was only subjected to laparotomy. After the reperfusion of 24 h, the mice were sacrificed. The blood samples were collected to detect the concentrations of serum creatinine (Scr) and blood urine nitrogen (BUN). Meanwhile, kidney cortex tissues were also collected for histological analysis and protein analysis. The experiments performed were approved by the Experimental Animal Welfare Ethics Committee of Qingdao University (No. 202105C5742202106034); all operations performed in animal experiments were in compliance with the ethical standards of the Chinese Association of Laboratory Animal Care.

### Detection of Renal Function

Scr and BUN levels were detected using Creatinine Assay Kit (C011-2-1) and Urea Assay Kit (C013-2-1) from the Nanjing Jiancheng Biological Engineering Institute. The collected blood samples were clotted at room temperature for 1 h and then centrifuged at 3,000 ×g for 15 min at 4°C to obtain serum. The serum samples were then transferred to clean tubes and immediately stored at -20°C for further detection. The samples were added to the reaction mixture and incubated at 37°C. The optical density (OD) values at 546 nm were measured at 5 and 10 min to calculate Scr concentrations. BUN was determined by applying the principle of the urease method, and BUN concentrations were calculated by measuring the OD values of the reaction mixture at 640 nm.

### Hematoxylin–Eosin Staining and Histopathological Analysis

The complete kidney samples from all treated groups were fixed in 10% formalin at 4°C for 24 h and embedded in paraffin. Then, samples were cut into 3-μm-thick sections. After dewaxing with xylene and hydration with different concentrations of ethanol, the sections were stained with hematoxylin and eosin (H&E). Blind-labeled sections were observed, and H&E staining images were captured using a light microscope (DP73, Olympus, Tokyo, Japan) under the magnifications of 400×. To evaluate the extent of tubular injury accurately, every kidney section was scored based on the visible damages in the tubules, including vacuolation, loss of brush border, tubular dilation, cast formation, and cell necrosis. A score of 0 indicated that there was no damage; one indicated < 25% damage; two indicated 25%-50% damage; three indicated 50%–75% damage; and four indicated > 75% damage. A minimum of 10 high magnification fields (400×) were scored for each of the mice. The average scores of all 10 fields were assigned as the tubular injury scores of mice in different treated groups.

### Terminal Deoxynucleotidyltransferase-Mediated dUTP Nick End Labeling

An *In Situ* Cell Death Detection Kit (Roche Applied Science, Indianapolis, IN, United States) was used for the assessment of apoptosis in renal tissues by a TUNEL assay according to the manufacturer’s instructions. TUNEL-positive cells were counted in 10 random high magnification fields (400×) of each section under a light microscope. The index of TUNEL-positive cells was obtained by calculating the ratio of TUNEL-positive nuclei to total nuclei in tubular epithelial cells.

### Immunohistochemistry

Immunohistochemistry was used for the identification of p-ERK and EGR1. The protocols were described as follows: blocking buffer: 3% BSA (G5001, Servicebio); primary antibody: anti-p-ERK1/2 antibody (ab201015, Abcam) and anti-EGR1 antibody (22008-1-AP, Proteintech) incubated overnight at 4°C; secondary antibody (E-AB-1034, Elabscience Biotechnology) was incubated for 50 min at room temperature. They were then stained using a DAB Kit (G1211, Servicebio). A light microscope was used for morphology assessment. Average optical density (AOD) values of p-ERK1/2 and EGR1 were analyzed using ImageJ software. AOD = integrated density/area.

### Measurement of Cyt C

Mouse Cytochrome C ELISA Kit (CSB-E08532m, Cusabio, Wuhan) was used for the detection of Cyt C. Kidney tissues were collected from different treated groups. After being homogenized in 1 × PBS, homogenates were centrifuged for 5 min at 5000 ×g, 4°C. The supernatant was removed and assayed immediately. According to the manufacturer’s instructions, the standard curve was plotted by the mean absorbance of each standard with its concentration using CurveExpert Professional software (version 1.6.5). Then, the best-fit curve was selected for interpolations.

### Bioinformatics

The raw data (TAR OF CEL) of GSE52004 dataset (Affymetrix Mouse Gene 1.0 ST Array) were downloaded from GEO DataSets (Gene Expression Omnibus, http://www.ncbi.nlm.nih.gov/geo/). The AKI samples of GSM116, GSM117, GSM118, and GSM119 and sham samples of GSM120 and GSM121 were used for analysis, which were isolated from the kidneys of C57BL/6 mice. The raw data were then quality controlled and normalized by a robust multi-array averaging algorithm (Giorgi et al., 2010). Differential expression analysis was performed by the Linear Models for Microarray Analysis (*Limma*) package (Ritchie et al., 2015). The *p*-value was adjusted using the *B*enjamini and Hochberg method. Absolute log_2_FC > 1 with adjusted *p*-value (adj. *P*) < 0.05 was considered the threshold of differentially expressed genes (DEGs). Gene-set enrichment analysis (GSEA) was performed using the *clusterProfiler* (Yu et al., 2012) package. In brief, FDR < 0.05 with an absolute normalized enrichment score (NES)>1 was regarded as significant enrichment. *RcisTarget* (Aibar et al., 2022) was applied to identify enriched transcription factor (TF)-binding motifs and candidate TFs (Aibar et al., 2017). Motifs were annotated to TFs based on the pathway enrichment analysis, and those with NES ≥ 3 were retained. (Aibar et al., 2022).

We acquired protein–protein interaction (PPI) networks from the STRING database (https://www.string-db.org/) and selected the medium confidence (0.4) as the minimum required interaction score for screening the interaction among DEGs. Cytoscape software (version 3.8.0) visualized PPI networks, and *cytoHubba*, a plugin of Cytoscape, was used to calculate node scores of genes by the maximal clique centrality (MCC) method (Chin et al., 2014) which has a better performance on the precision of predicting essential proteins. Genes with top 50% of the scores were regarded as hub genes.

### Molecular Docking

AutoDock (version 4.2.6) was used for molecular docking of tilianin and hub gene-encoded proteins (Forli et al., 2016). The PDB format files of proteins were downloaded from the RCSB Protein Data Bank (https://www1.rcsb.org/). The SDF format file of tilianin was obtained from the NCBI PubChem Compound database (https://www.ncbi.nlm.nih.gov/pccompound/), and then Open Babel GUI (O'Boyle et al., 2011) (version 2.4.1) was used to convert the SDF format file into PDB format file. After removal of solvent molecules and ligand, hydrogenation, electron, and other operations, the files of hub gene-encoded proteins set as receptors were prepared. Then, a PDBQT format file of tilianin set as the ligand was established, and molecular docking was performed subsequently. AutoDockTools (version 1.5.7) was used to analyze the results, and PyMOL (version 2.4.1) was used for visual simulation.

### Quantitative Real-Time PCR (qRT-PCR)

Total RNA of kidney tissues was isolated with RNAex pro reagent (Accurate Biotechnology, Hunan), and 1 mg RNA of each sample was reversely transcribed into cDNA for detection of mRNA expression. Then, qRT-PCR was performed in a CFX96 Touch Real-Time PCR System (Bio-Rad, Hercules, CA, United States) using a SYBR ExScript qRT-PCR Kit (TaKaRa Bio) with the following conditions: at 95°C for 1 min followed by 40 cycles at 95°C for 15 s, 60°C for 15 s, and 72°C for 45 s. Primer sequences used in the experiment are shown in Table 1. β-Actin was considered as a reference gene to normalize mRNA quantity, and the 2^−ΔΔCT^ method was used for the calculation of relative mRNA expression.

**TABLE 1:** Primer subsequences used in the article.

Genes	Forward primer	Reverse primer
m-β-Actin	ACG​GCC​AGG​TCA​TCA​CTA​TTG	AGA​GGT​CTT​TAC​GGA​TGT​CAA​CGT
m-KIM-1	CAG​GGA​AGC​CGC​AGA​AAA​A	GGA​AGG​CAA​CCA​CGC​TTA​GA
m-Caspase-3	GAG​GAG​ATG​GCT​TGC​CAG​AA	CTT​GTG​CGC​GTA​CAG​CTT​CA
m-BCL2	CGT​CGC​TAC​CGT​CGT​GAC​TT	CCC​CAC​CGA​ACT​CAA​AGA​AG
m-BCL2L1	GAG​AGG​CAG​GCG​ATG​AGT​TT	CGA​TGC​GAC​CCC​AGT​TTA​CT
m-BAX	CCA​AGA​AGC​TGA​GCG​AGT​GTC​T	TGA​AGT​TGC​CAT​CAG​CAA​ACA
m-BAD	GAG​GAG​CTT​AGC​CCT​TTT​CGA	TTT​GTC​GCA​TCT​GTG​TTG​CA
m-EGR1	GCAGCGGCGGTAATAGCA	CTC​CAC​CAT​CGC​CTT​CTC​AT

### Western Blot Analysis

Kidney tissues collected from mice were homogenized in RIPA lysis buffer (Elabscience Biotechnology, Wuhan) and PMSF protease inhibitor (Elabscience Biotechnology, Wuhan) for 30 min and then centrifuged at 15,000 ×g for 30 min at 4°C. The supernatants were transferred into clean tubes, and protein concentrations were measured using the BCA protein concentration assay kit (Elabscience Biotechnology, Wuhan). Then, 5× SDS loading buffer (P0015, Beyotime) was added to the supernatants and heated at 95°C for 15 min. Proteins equal in quality were separated in 10% SDS-PAGE and then transferred onto PVDF membranes of 0.45 μm (Millipore, Germany). The membranes were blocked with 5% skimmed milk for 1 h at room temperature and then incubated overnight with primary antibodies against β-actin (E-AB-20058, Elabscience Biotechnology), extracellular regulated MAP kinase1/2 (ERK1/2, ab184699, Abcam), phospho-ERK1/2 (p-ERK1/2, ab201015, Abcam), early growth response 1 (EGR1, 22008-1-AP, Proteintech), BCL2 apoptosis regulator (BCL2, E-AB-22004, Elabscience Biotechnology), BCL2-associated X apoptosis regulator (BAX, E-AB-10049, Elabscience Biotechnology), BCL2-like 1 (BCL2L1, E-AB-40057, Elabscience Biotechnology), BCL2-associated agonist of cell death (BAD, AF7927, Affinity), and caspase-3 (ab184787, Abcam). The membranes were incubated with secondary antibodies (goat anti-mouse IgG, E-AB-1035, Elabscience Biotechnology; goat anti-rabbit IgG, E-AB-1034, Elabscience Biotechnology) for 1 h at room temperature after being washed three times with the phosphate buffer solutions with Tween-20 (PBST) and detected using the excellent chemiluminescent substrate (ECL) detection kit (E-IR-R307, Elabscience Biotechnology). The chemiluminescence gel imaging system was used to detect the Western blot bands, and ImageJ software was used for band scanning. The ratio of the target protein to the reference protein was used to correct errors.

### Statistical Analysis

All data were expressed as means ± standard deviation (SD). Statistical significance was analyzed using ANOVA followed by the Bonferroni *post hoc* test. A value of *p* < 0.05 was considered statistically significant.

## Results

### Tilianin Improved Kidney Function of I/R-Induced AKI Mice

**FIGURE 2:**
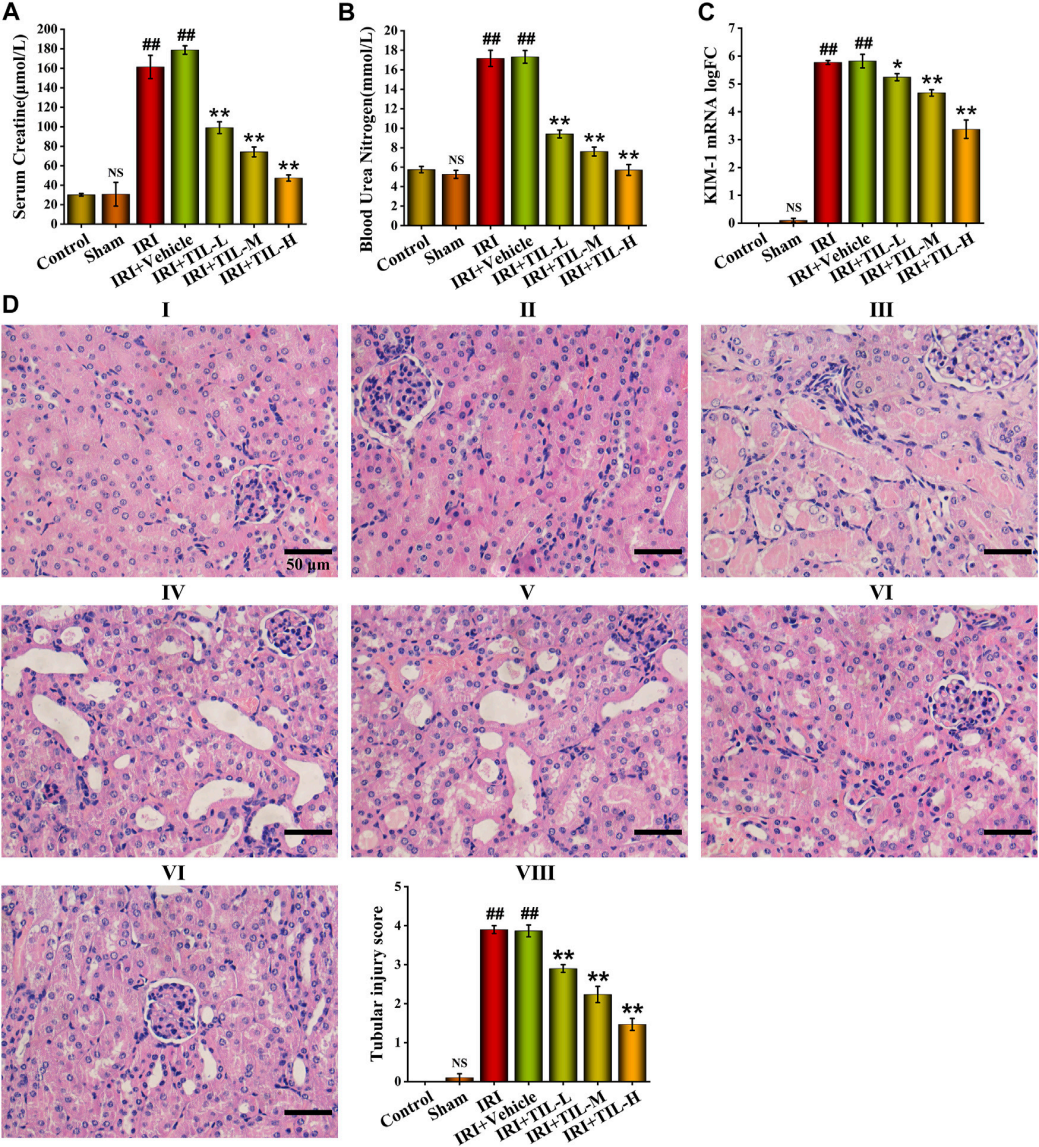
Tilianin improved kidney function of I/R-induced AKI mice. **(A,B)** Quantitative analysis of Scr and BUN in different groups, as indicated. **(C)** Quantitative analysis of KIM-1 mRNA levels determined by qRT-PCR in different groups, as indicated. **(D)** H&E staining images under the magnifications of 400× showed the extent of tubular injury, and pathological score was quantified based on the standard described in “Methods and Materials.” Scale bar, 50 μm (Ⅰ: control group; Ⅱ: sham group; Ⅲ: IRI group; Ⅳ: IRI+vehicle group; Ⅴ: IRI+TIL-L group; Ⅵ: IRI+TIL-M group; Ⅶ: IRI+TIL-H group; Ⅷ: quantitative analysis of tubular injury scores). IRI: ischemia–reperfusion injury group; IRI+TIL-L: IRI mice treated with tilianin 5 mg/kg group; IRI+TIL-M: IRI mice treated with tilianin 10 mg/kg group; and IRI+TIL-H: IRI mice treated with tilianin 15 mg/kg group. *n* = 3 per group. All data are shown as mean ± SD. ^*##*^ *p* < 0.01 *versus* control*;* ^*NS*^ *P* > 0.05 *versus* control; *p < 0.05, **p < 0.01 *versus* IRI.

To investigate whether tilianin improves kidney function after AKI, we employed a mice I/R-induced AKI model and measured Scr and BUN levels as well as the renal pathological changes (Figure 1B). As a result, the IRI group exhibited elevated Scr and BUN levels compared to control groups, while tilianin reduced both of the levels in a dose-dependent manner (Figures 2A,B), as well as the KIM-1 mRNA expression, indicating that tilianin alleviates kidney injury (Figure 2C). Furthermore, HE staining showed severe tubular injuries in the IRI group (Figure 2D), but tilianin administration reduced the tubular injury score to approximately half that was observed in the IRI group. These results all suggested that tilianin protected kidney function *in vivo*.

### Tilianin Reduced Apoptosis in Mice After I/R-Induced AKI

**FIGURE 3:**
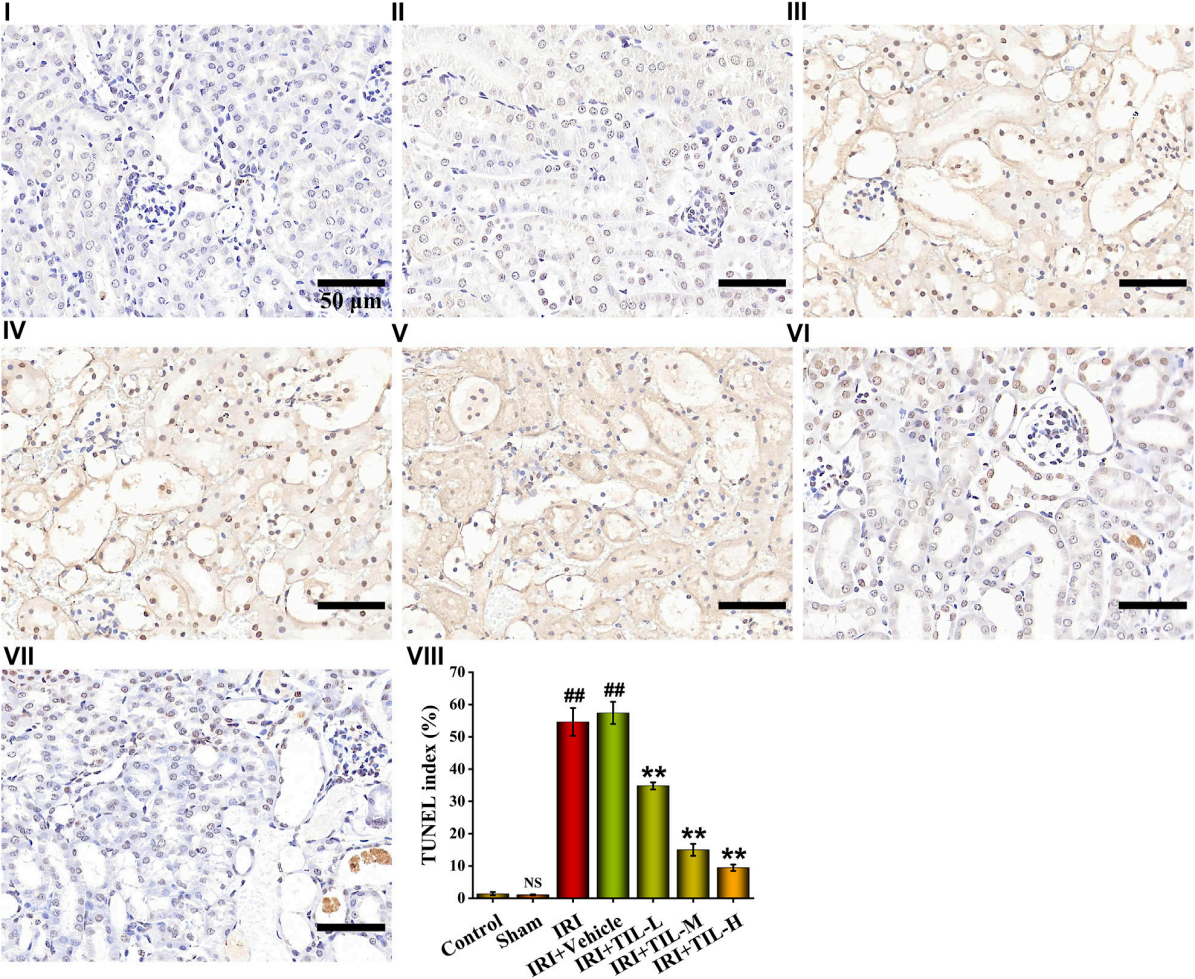
TUNEL assessment of different treatment groups. TUNEL images of kidney sections under the magnifications of 400× showed the number of apoptotic cells in the different groups, which were stained with blue and brown. TUNEL index were proportions of TUNEL-positive nuclei to total nuclei in RTECs of mice. Scale bar, 50 μm (Ⅰ: control group; Ⅱ: sham group; Ⅲ: IRI group; Ⅳ: IRI+vehicle group; Ⅴ: IRI+TIL-L group; Ⅵ: IRI+TIL-M group; Ⅶ: IRI+TIL-H group; and Ⅷ: statistical results of the TUNEL index). n =3 per group. All data are shown as mean ± SD. ^*##*^ *p* < 0.01 *versus* control*;* ^*NS*^ *P* > 0.05 *versus* control. **p < 0.01 *versus* IRI.

**FIGURE 4:**
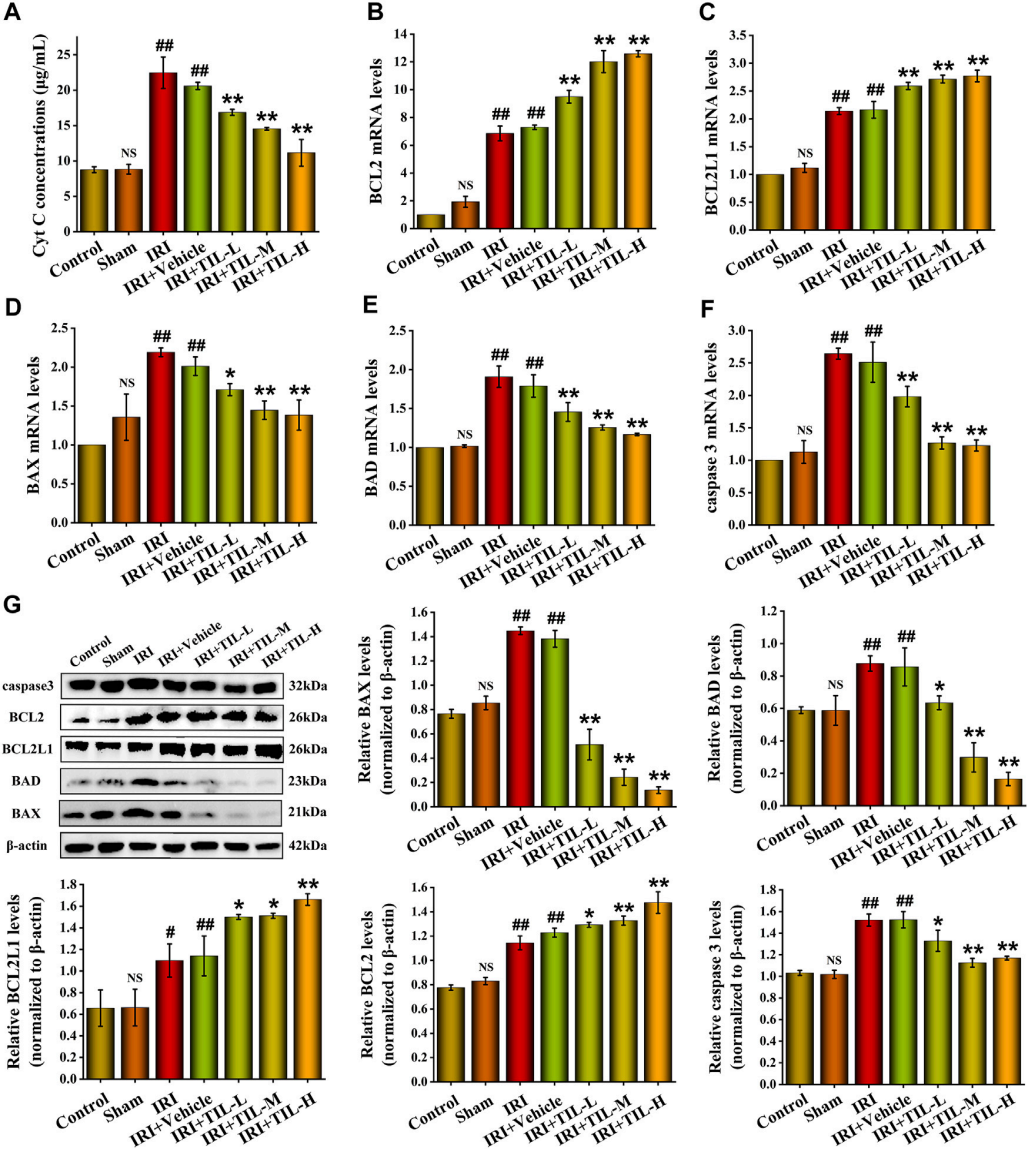
Tilianin reduced apoptosis in mice after I/R-Induced AKI. **(A)** Concentrations of Cyt C released into the cytoplasm from different groups, as indicated. **(B,C)** Relative mRNA levels of anti-apoptotic genes BCL2 and BCL2L1 determined by qRT-PCR in different groups, as indicated. **(D–F)** Relative mRNA levels of pro-apoptotic genes BAX, BAD, and caspase-3 determined by qRT-PCR in different groups, as indicated. **(G)** Protein expression and statistical results of apoptosis-related genes BAX, BAD, BCL2L1, BCL2, and caspase-3 in different groups, as indicated. n=3 per group. All data are shown as mean ± SD. ^*#*^ *p* < 0.05, ^*##*^ *p* < 0.01 *versus* control*;* ^*NS*^ *P* > 0.05 *versus* control; *p < 0.05, **p < 0.01 *versus* IRI.

TUNEL assessment showed that the tubular injury induced by I/R raised the number of apoptosis cells in RTECs when compared with the control groups, while tilianin improved the degree of apoptosis significantly, especially in the tilianin high-dose treatment group (Figure 3). Tilianin pretreatment reduced the release of Cyt C (Figure 4A), along with the increasing expressions of anti-apoptotic genes BCL2 and BCL2L1 in both mRNA and protein levels, indicating the anti-apoptotic effect of tilianin (Figures 4B,C,G). Conversely, the expressions of pro-apoptotic genes, BAX, BAD, and executioner caspase-3, were decreased by tilianin (Figures 4D–G). Hence, tilianin has beneficial effects on I/R-induced apoptosis in the mitochondrial pathway.

### Bioinformatics Identified Apoptosis-Related Hub Differential Transcription Factors

**FIGURE 5:**
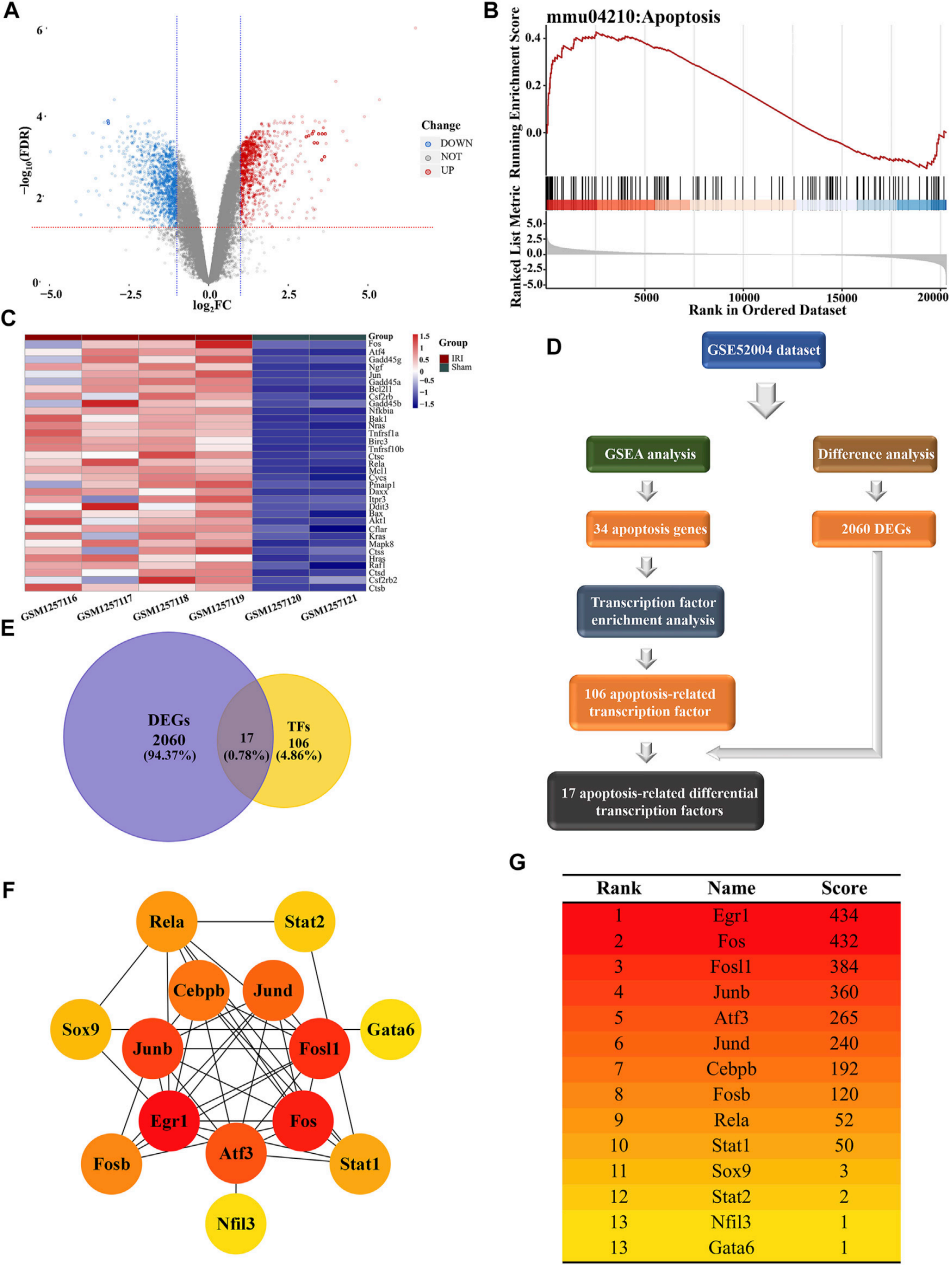
Bioinformatics analysis conducted for apoptosis-related differential transcription factors. **(A)** Volcano plot of GSE52004 illustrated the DEGs identified by quantitative analysis. The red horizontal dashed line represents -log_10_ (FDR=0.05), and the two blue vertical dashed lines represented log_2_FC=-1 and log_2_FC=1, respectively. Among the DEGs, 943 upregulated genes were marked in red dots, while 1,117 downregulated genes were marked in blue dots. **(B)** GSEA of microarray data shows the enrichment plot of the apoptosis pathway for sets of genes in the GSE52004 dataset. NES=1.4913. **(C)** Heatmap showed the expression of 34 apoptosis-related genes in the GSE52004 dataset. The color of each section was proportional to the significance of changes in apoptosis (red indicated increase; blue indicated decrease). **(D)** Flow chart exhibits the discovery process of apoptosis-related differentially expressed TFs. **(E)** Venn diagram shows the intersection between DEGs and TF datasets, which meant the genes from the intersection were differentially expressed transcription factors. **(F)** Network of 14 differentially expressed TFs associated with apoptosis visualized by Cytoscape. The different levels of color were proportional to the significance of genes in the network. **(G)** Scores and rankings of 14 apoptosis-related genes calculated by the MCC method are described in “Methods and Materials” *via* Cytoscape. The color of the genes corresponded to the color in the network.

To identify gene enrichment in the apoptosis pathway after I/R-induced AKI, we analyzed the GSE52004 dataset between sham and AKI groups. Differential expression analysis showed that there were 943 upregulated genes and 1,117 downregulated genes after AKI (Figure 5A). The GSEA showed that 34 genes, including FOS, ATF4, and Gadd45g, were involved in the apoptosis pathway, and most genes were upregulated after I/R-induced AKI (Figure 5B). The expressions of these genes in AKI and sham groups were presented as a heatmap (Figure 5C). TF enrichment analysis predicted 106 TFs with 162 motifs (NES ≥ 3), of which only 17 TFs were differentially expressed after I/R-induced AKI out of 106 genes (Figures 5D,E). In order to identify the genes that were at the core of transcriptional regulation, a PPI network was established for 17 TFs. Among these, 14 genes have interactions with each other (Figure 5F). Then, these genes were scored using the MCC method and ranked by Cytoscape. Finally, according to the ranking result, 7 genes were selected as hub TFs (Figure 5G).

### Tilianin Had the Potential to Regulate Apoptosis-Related Transcription Factors After I/R-Induced AKI

**FIGURE 6:**
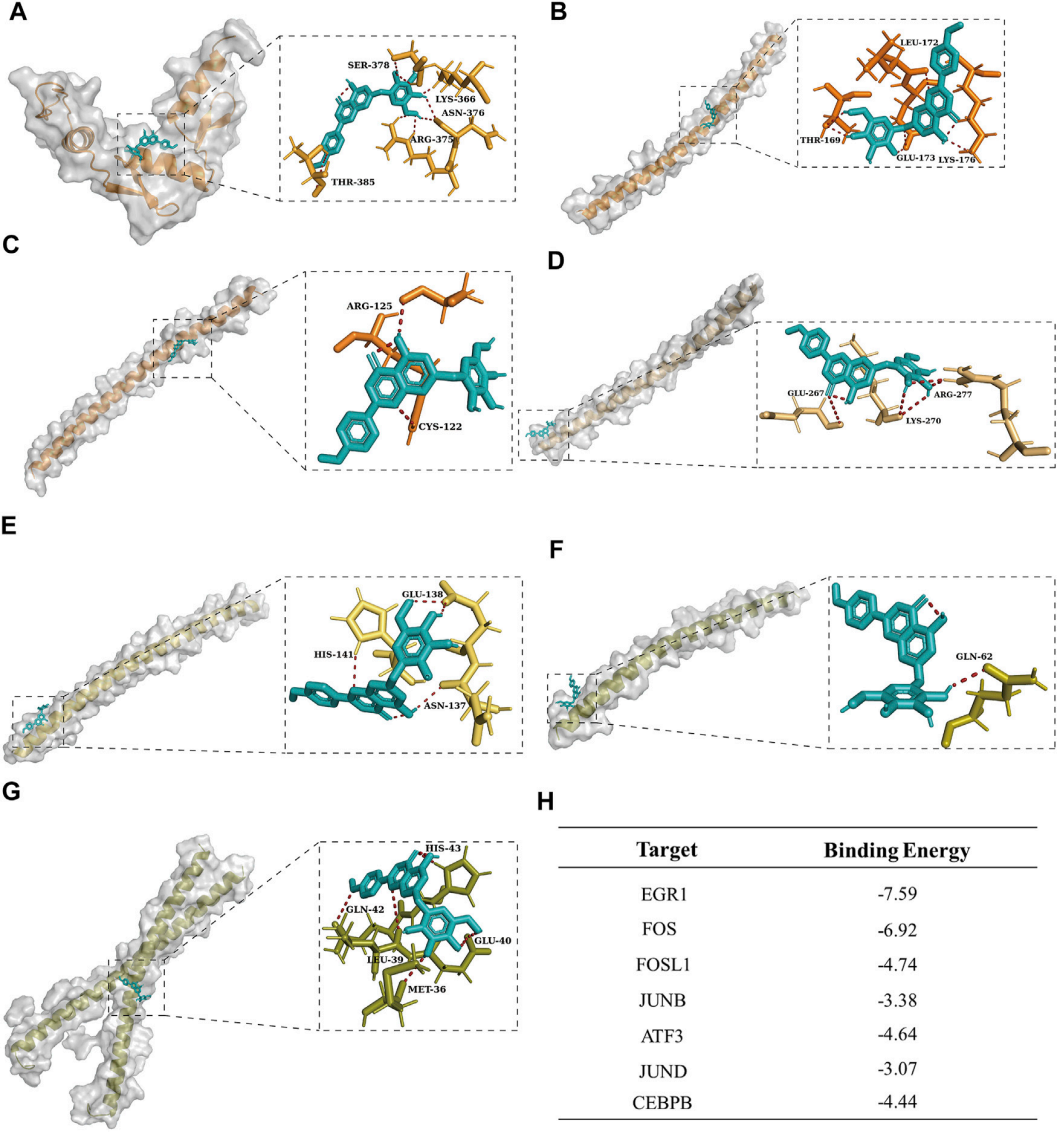
Molecular docking visualization results between tilianin and corresponding targets. Tilianin (ligand) was bound to the surface of seven hub genes (receptor) with amino acid residues of receptors. Tilianin was shown as the red stick structure, and the receptors were painted in different colors. Hydrogen bonds are shown as dotted lines, as indicated. **(A)** EGR1-tilianin, **(B)** FOS-tilianin, **(C)** FOSL1-tilianin, **(D)** JUNB-tilianin, **(E)** ATF3-tilianin, **(F)** JUND-tilianin, and **(G)** CEBPB-tilianin. **(H)** Binding energy between tilianin and targets.

Given the wide range of applications and pharmacological activity of tilianin, we virtually docked tilianin with seven hub TFs by AutoDock (Figures 6A–G). As a result, the binding energy between tilianin and seven targets was low (Figure 6H), suggesting that tilianin regulates apoptosis-related TFs potentially. Considering that EGR1 gained the lowest binding energy, tilianin plays a role in the transcriptional regulation of EGR1 after I/R-induced AKI.

### The Potential of Tilianin to Regulate the Transcriptional Activation of EGR1 Was Found

**FIGURE 7:**
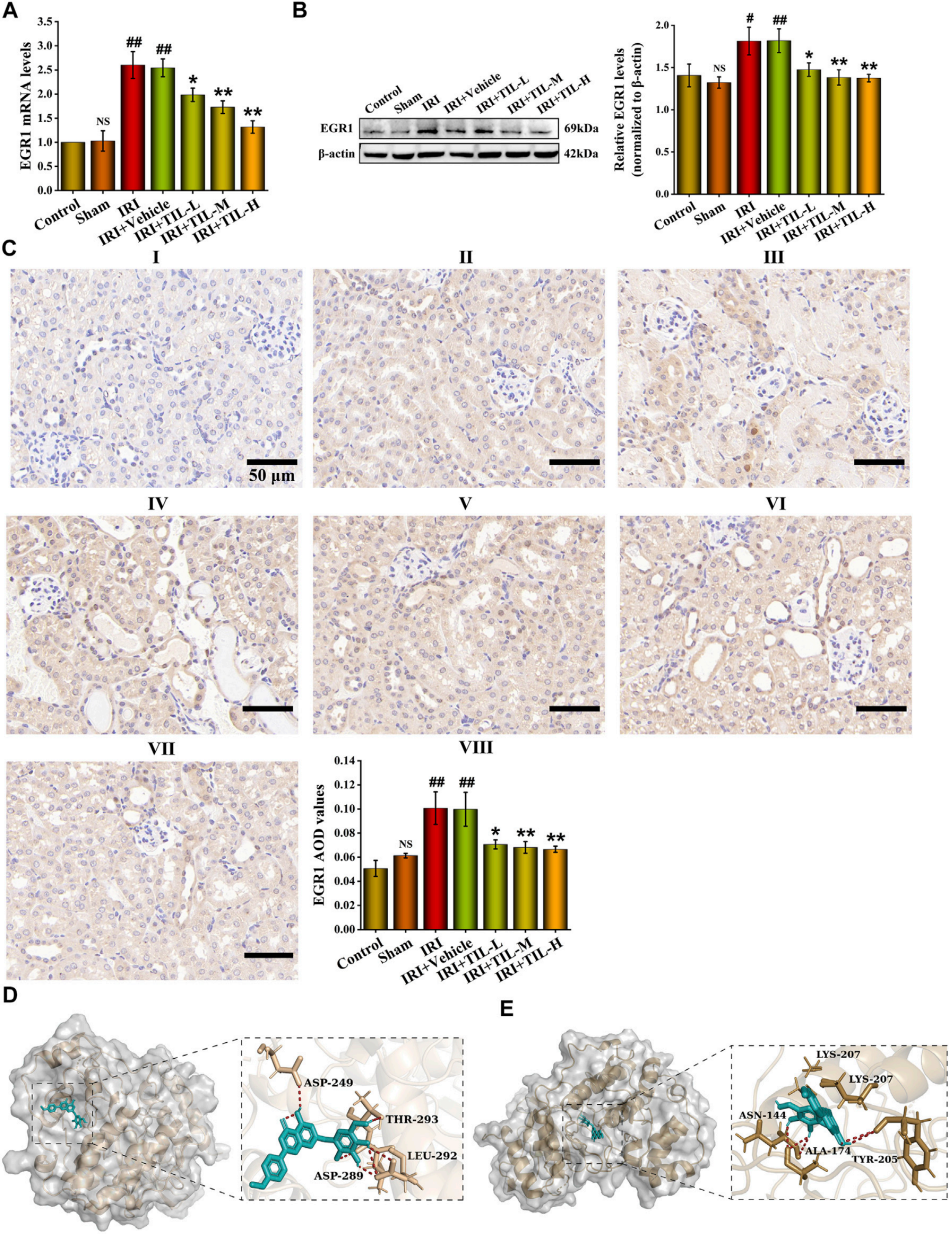
Potential of tilianin to regulate the transcriptional activation of EGR1 was found. **(A)** Relative mRNA levels of EGR1 determined by qRT-PCR in different groups, as indicated. **(B)** Protein expression and statistical results of EGR1 in different groups, as indicated. *n* = 3 per group. **(C)** Immunohistochemistry images of EGR1 under the magnifications of 400 × show the location and quantification of EGR1 in the mouse kidney after I/R-induced AKI. Scale bar, 50 μm (Ⅰ: control group; Ⅱ: sham group;Ⅲ: IRI group; Ⅳ: IRI+vehicle group; Ⅴ: IRI+TIL-L group; Ⅵ: IRI+TIL-M group; Ⅶ: IRI+TIL-H group; and Ⅷ: statistical results of EGR1 AOD values). **(D,E)** Molecular docking visualization results of ERK-tilianin and p-ERK-tilianin. Tilianin is shown as the red stick structure, and the receptors are painted in different colors. Hydrogen bonds are shown as dotted lines, as indicated. All data are shown as mean ± SD. ^*#*^ *p* < 0.05, ^*##*^ *p* < 0.01 *versus* control*;* ^*NS*^ *P* > 0.05 *versus* control; *p < 0.05, **p < 0.01 *versus* IRI.

We next detected the expression of EGR1 after the treatment of tilianin. The mRNA levels of EGR1 in tilianin treatment groups decreased compared with the group of AKI (Figure 7A). In addition, the protein levels of EGR1 showed the same trend (Figures 7B,C). Based on the phenomenon of EGR1 expression decline after tilianin treatment, we virtually docked tilianin with ERK and p-ERK, which showed that the binding energy was -8.51 and -8.03 between them (Figures 7D,E). Consistent with previous studies, tilianin could regulate the activity of EGR1 after I/R-induced AKI by acting on ERK pathways whose downstream target was EGR1.

### Tilianin Attenuated Apoptosis After I/R-Induced AKI via the ERK/EGR1/BCL2L1 Pathway

**FIGURE 8:**
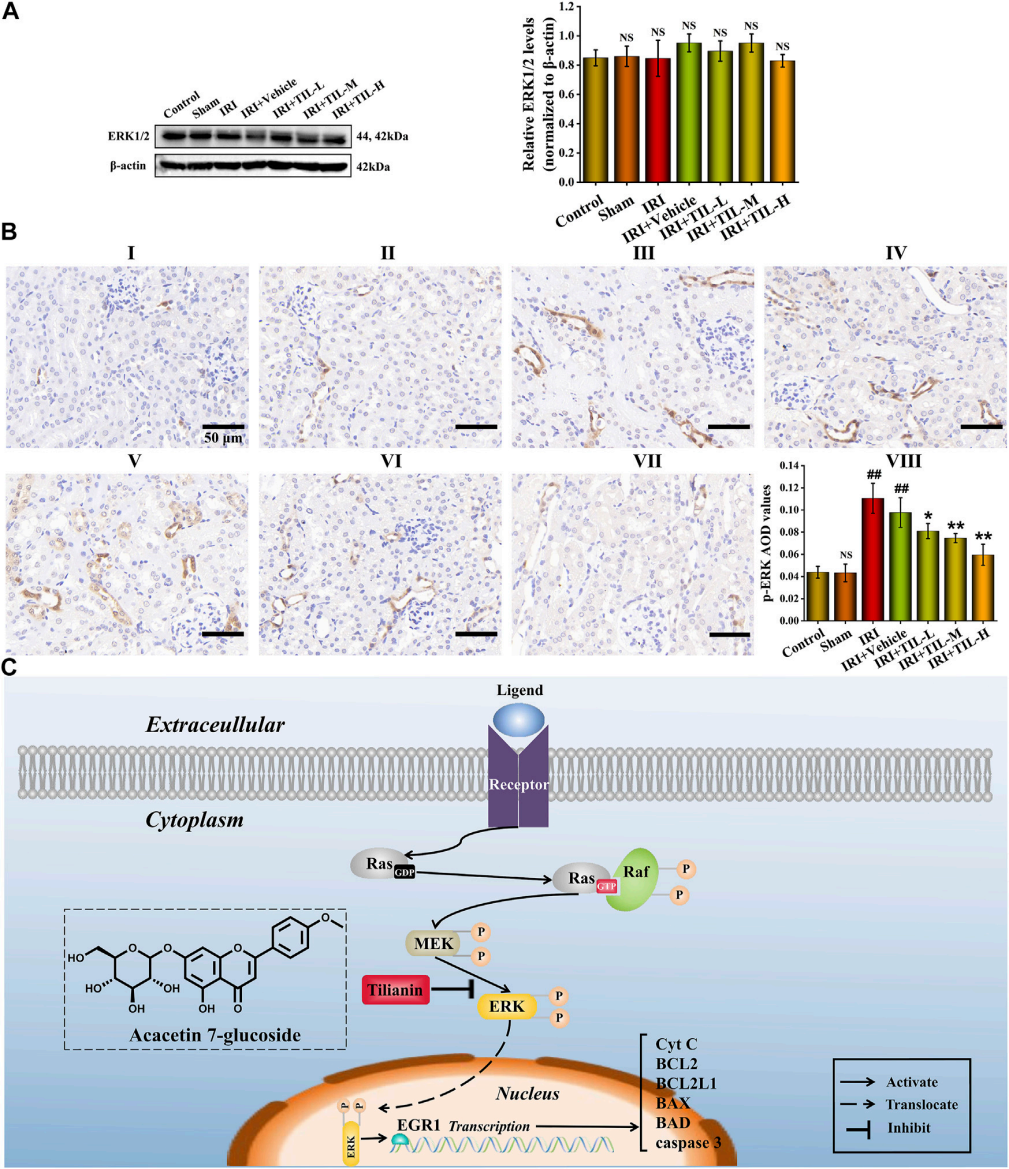
Tilianin attenuated apoptosis after I/R-induced AKI *via* the ERK/EGR1/BCL2L1 pathway. **(A)** Protein expression and statistical results of ERK1/2 in different groups, as indicated. *n* = 3 per group. **(B)** Immunohistochemistry images of p-ERK under the magnifications of 400× show the location and quantification of p-ERK in the mouse kidney after I/R-induced AKI. Scale bar, 50 μm (Ⅰ: control group; Ⅱ: sham group; Ⅲ: IRI group; Ⅳ: IRI+vehicle group; Ⅴ: IRI+TIL-L group; Ⅵ: IRI+TIL-M group; Ⅶ: IRI+TIL-H group; and Ⅷ: statistical results of p-ERK AOD values). **(C)** Schematic figure illustrated that once RTECs suffered from external stimulation, the ERK pathway was activated, resulting in increased phosphorylation of ERK1/2 and its translocation. Tilianin alleviated apoptosis after I/R-induced AKI *via* reducing the phosphorylation level of ERK1/2 and transcriptional activation of EGR1. All data are shown as mean ± SD. ^*##*^ *p* < 0.01 *versus* control*;* ^*NS*^ *P* > 0.05 *versus* control or IRI; *p < 0.05, **p < 0.01 *versus* IRI.

To explore the mechanism of tilianin for attenuating apoptosis after I/R-induced AKI, the protein levels of ERK1/2 and p-ERK were analyzed by Western blot. As expected, the phosphorylation level of ERK1/2 was significantly increased after AKI. In contrast, pretreatment with tilianin reduced the phosphorylation level of ERK1/2 (Figures 8A,B). We found that the expression level of EGR1 was consistent with that of p-ERK, which meant the reduction of ERK1/2 phosphorylation decreased the expression of EGR1 after the pretreatment of tilianin. The pretreatment of tilianin decreased the locations of p-ERK in the nucleus (Figure 8B), and EGR1 participated in the transcriptional regulation of downstream target BCL2L1 based on the motif in the TF enrichment analysis (Supplementary Figure S1). Therefore, tilianin could attenuate apoptosis in the mitochondrial pathway via the ERK/EGR1/BCL2L1 pathway.

## Discussion

Natural products have been extensively considered an important regimen for treating refractory kidney diseases (Chen et al., 2018; Miao et al., 2021; Soriano-Castell et al., 2021; Yang and Wu, 2021; Zhao et al., 2021). In this study, we found for the first time that tilianin protected mice from I/R-induced AKI, especially it inhibited cell apoptosis in the mitochondrial pathway. Mechanistically, we demonstrated the ERK/EGR1/BCL2L1 pathway mediated by tilianin using a series of bioinformatics methods for predicting and *in vivo* experiments for validation (Figure 8C). Microarray analysis and transcriptional enrichment analysis identified EGR1 as a hub TF regulating cell apoptosis after I/R-induced AKI. In addition, AutoDock speculated tilianin’s potential to regulate apoptosis-related hub TFs, and ERK signaling pathway was also involved in the anti-apoptotic pathway. Consistent with the results of *in vivo* experiments, we uncovered that tilianin inhibited cell apoptosis in a mitochondrial pathway by attenuating ERK pathway activation and downregulating the expression of EGR1. Therefore, our present study identified tilianin as a promising therapeutic agent against I/R-induced AKI.

Hypovolemia, hypotension, and heart failure are all common causes of transient ischemia, which contributed to AKI easily and explained for nearly one-third of patients requiring renal replacement therapy (Havasi and Borkan, 2011; Deng et al., 2020; Sabapathy et al., 2020). In I/R-induced AKI, proximal tubular epithelial cells were highly susceptible to injury, and apoptosis played a critical role in this process (de Ponte et al., 2021; Tonnus et al., 2021). Mitochondria are a key site for integrating pro- and anti-apoptotic proteins in renal cells. When proximal tubular epithelial cells suffered from the stress of injury, pro-apoptotic proteins, including BAX and BAD, bound to the mitochondrial membrane, and this process led to the increased membrane permeability. Cyt C was then released into the cytoplasm and activated “apoptosis executioner” caspase families, which promoted the occurrence of apoptosis. Thus, agents with the ability to inhibit the mitochondrial apoptosis pathway and protect against I/R-induced AKI appeared to be significant. In previous studies, tilianin has been proved to attenuate myocardial I/R-induced injury and mediate neuroprotection against ischemic injury (Wang et al., 2017; Jiang et al., 2019). However, no study focused on the effects of tilianin for I/R-induced AKI. Our study uncovered the nephron protection and anti-apoptotic effect of tilianin against I/R-induced AKI for the first time. Mechanically, both signal transduction pathway and TFs were focused in our study. EGR1 was an immediate early gene and was involved in growth, differentiation, apoptosis, neurite outgrowth, and wound healing (Li et al., 2019). In kidney diseases, EGR1 was observed in the proximal tubule in response to hypoxic stimuli, and silencing of EGR1 could alleviate the injury in diabetic kidney disease and protect from renal inflammation and fibrosis (Sun et al., 2014; Miyamoto et al., 2016; Hu et al., 2020). In our study, EGR1 was identified as a hub TF, and both mRNA and protein levels were observed to be upregulated in I/R-induced AKI. However, tilianin reduced its expression, and apoptosis levels were subsequently improved. TFs represent the convergence point of multiple signaling pathways in eukaryotic cells (Papavassiliou and Papavassiliou, 2016). Most often, EGR1 was rapidly activated via extracellular signal-regulated kinases (Brennan et al., 2018). AutoDock showed that tilianin had lower binding energy with ERK and p-ERK. Then, we detected the expression of ERK and p-ERK and found that tilianin decreased the phosphorylation of the ERK pathway, which resulted in declined translocation and localization of p-ERK in the nucleus. Active ERKs regulate phosphorylation of many cytoplasmic and nuclear targets, including apoptosis, autophagy, and senescence (Ramos, 2008). Yong et al. found that the activation of ERK1/2 was essential for the cisplatin-induced apoptosis of renal epithelial cells (Kim et al., 2005). Wang et al. (2016) described the protection of U0126 against hypoxia/reoxygenation-induced myocardium apoptosis and autophagy via the MEK/ERK/EGR-1 pathway. Therefore, our findings provided novel insights into the involvement of ERK pathway and EGR against I/R-induced renal cell apoptosis.

Different from previous studies, our research explored more comprehensive molecular mechanisms. Many bioinformatics methods were conducted for the speculation of the underlying molecular mechanism. From execution proteins to signal transduction pathway, the regulating pathway for tilianin against I/R-induced renal cell apoptosis in AKI was fully demonstrated. In addition, tilianin was used in *in vivo* experiments for the investigation of dose-dependent properties. It should be noted that ERK1/2 and EGR1 may not be the only molecules involved in tilianin-mediated renoprotection. It could not be convinced for the binding between tilianin and ERK1/2 or p-ERK. But it is believed that tilianin reduced the phosphorylation of the ERK pathway, which further decreased the effects on downstream regulations. Interestingly, active ERKs had been found to be localized to mitochondrial membranes, which provided the possibility for direct regulation of apoptosis-associated proteins. (Nowak et al., 2006; Zhuang et al., 2007). Also, tilianin still has other effects such as antioxidant and anti-inflammatory, but in this study only the anti-apoptotic effects and associated mechanisms of tilianin were described and demonstrated. The exploration for other effects of tilianin against I/R-induced AKI will be fully described in our future research study. Despite these limitations, we believed our findings provided a novel comprehension of the anti-apoptotic properties of tilianin against I/R-induced AKI.

In conclusion, our study provided novel evidence indicating that tilianin could protect mice against I/R-induced AKI by improving mitochondrial pathway apoptosis, a process by which tilianin at least partially reduces phosphorylation of the ERK pathway and further decreases the transcriptional activation of EGR1. Pro-apoptotic proteins, including BAX, BAD, and caspase-3, were downregulated, while anti-apoptotic proteins like BCL2 and BCL2L1 were upregulated. This resulted in the less release of Cyt C. Collectively, these findings suggested that tilianin might be a new potential agent for the therapy of I/R-induced AKI.

## Data Availability

Publicly available datasets were analyzed in this study. These data can be found at: GEO DataSets (Gene Expression Omnibus) GSE52004.
