# Supplementary material for: Tilianin Reduces Apoptosis via the ERK/EGR1/BCL2L1 Pathway in Ischemia/Reperfusion-Induced Acute Kidney Injury Mice
Source: Front Pharmacol. 2022 Jun 3;13:862584. doi: 10.3389/fphar.2022.862584 (PMC9204490; doi:10.3389/fphar.2022.862584)
Supplement: Supplementary file 1 [file Image1.pdf]

Supplementary Material

1 Supplementary Figures and Tables

	logo	motif	NES	AUC	TF_highConf
1	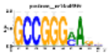	predrem__nrMotif909	5.27	0.16	
2	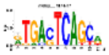	cisbp__M3617	5.25	0.16	Nfe2
3	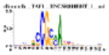	dbcorrdb__TAF1__ENCSR000BHT_1__m1	4.93	0.152	Taf1
4	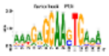	factorbook__PU1	4.83	0.149	Irf4; Spi1
5	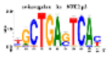	swissregulon__hs__NFE2.p2	4.81	0.149	Nfe2
6	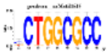	predrem__nrMotif1515	4.77	0.148	
7	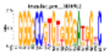	transfac_pro__M06912	4.72	0.147	Zfp236
8	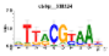	cisbp__M0324	4.71	0.146	
9	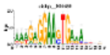	cisbp__M4489	4.59	0.144	Spi1
10	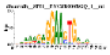	dbcorrdb__SPI1__ENCSR000BGQ_1__m1	4.50	0.141	Spi1
11	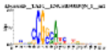	dbcorrdb__TAF1__ENCSR000BQN_1__m1	4.47	0.141	Taf1
12	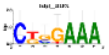	hdpi__H1FX	4.44	0.14	H1fx
13	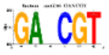	fantom__motif56_GANCGT	4.43	0.14	
14	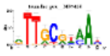	transfac_pro__M07414	4.40	0.139	
15	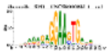	dbcorrdb__SPI1__ENCSR000BIJ_1__m1	4.29	0.136	Spi1
16	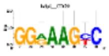	hdpi__CD59	4.25	0.135	Cd59a; Cd59b
17	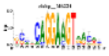	cisbp__M6224	4.22	0.134	
18	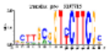	transfac_pro__M07715	4.20	0.134	
19	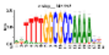	cisbp__M5359	4.18	0.133	E2f3
20	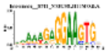	hocomoco__SPI1_MOUSE.H11MO.0.A	4.15	0.133	Spi1

21	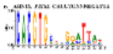	taipale tf pairs ARNTL PITX1 CACGTGNNNRGATTAN CAP repr	4.13	0.132	Arntl; Pitx1
22	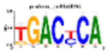	predrem nrMotif196	4.13	0.132	
23	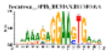	hocomoco__SPIB_HUMAN.H11MO.0.A	4.12	0.132	Spib
24	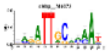	cisbp__M6173	4.11	0.132	Cebpg
25	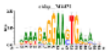	cisbp__M4475	4.10	0.132	Spi1
26	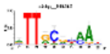	cisbp__M0315	4.09	0.131	Cebpb
27	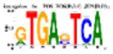	swissregulon__hs__FOS_FOS_B_L1__JUN_B_D_.p2	4.09	0.131	Fos; Fosb; Fosl1; Junb; Jund
28	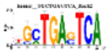	homer__TGCTGAGTCA_Bach2	4.06	0.131	Bach2
29	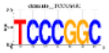	elemento__TCCCGGC	4.06	0.131	
30	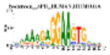	hocomoco SPI1 HUMAN.H11MO.0.A	4.05	0.13	Spi1
31	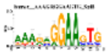	homer AAAGRGGAAGTG SpiB	4.04	0.13	Spib
32	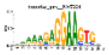	transfac pro M07224	4.01	0.129	Spi1
33	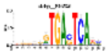	cisbp M4526	4.00	0.129	Smarcc1
34	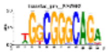	transfac_pro__M05885	3.97	0.128	Zfp872
35	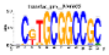	transfac_pro__M06035	3.91	0.127	
36	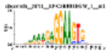	dbcorrdb__SPI1__ENCSR000BGW_1__m1	3.91	0.127	Spi1
37	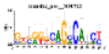	transfac_pro__M00733	3.9	0.127	Smad4
38	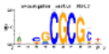	swissregulon__sacCer__RSC3	3.89	0.126	
39	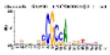	dbcorrdb SAP30 ENCSR000AQJ 1 m1	3.86	0.126	Sap30
40	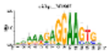	cisbp M1907	3.85	0.126	Spi1

41	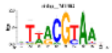	cisbp__M1402	3.82	0.125	
42	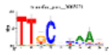	transfac_pro__M03571	3.82	0.125	Cebpd
43	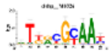	cisbp__M0326	3.8	0.124	
44	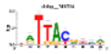	cisbp__M0316	3.8	0.124	Nfil3
45	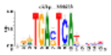	cisbp__M4623	3.78	0.124	Junb
46	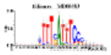	tfdimers__MD00383	3.78	0.124	Stat1; Tcf7l2
47	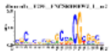	dbcorrdb__E2F6__ENCSR000EWJ_1__m2	3.78	0.124	E2f6
48	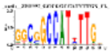	taipale_cyt_meth__ZNF597_GGCGGCCATYTTGN_FL_meth_repr	3.78	0.124	Zfp597
49	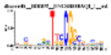	dbcorrdb__RBBP5__ENCSR000AQI_1__m1	3.77	0.124	Rbbp5
50	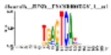	dbcorrdb__JUND__ENCSR000EGN_1__m1	3.77	0.124	Jund
51	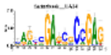	factorbook__UA14	3.76	0.123	
52	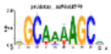	predrem__nrMotif970	3.75	0.123	
53	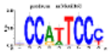	predrem__nrMotif162	3.75	0.123	
54	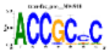	transfac_pro__M04918	3.74	0.123	Egr1
55	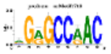	predrem__nrMotif1718	3.74	0.123	
56	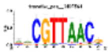	transfac_pro__M07564	3.73	0.123	
57	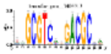	transfac_pro__M07713	3.72	0.122	Foxn2; Foxn3
58	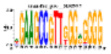	transfac_pro__M05997	3.72	0.122	Zfp64
59	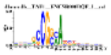	dbcorrdb__TAF1__ENCSR000BQF_1__m1	3.71	0.122	Taf1
60	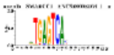	dbcorrdb__SMARCC1__ENCSR000EDM_1__m1	3.71	0.122	Smarcc1

Supplementary Figure 1. Motifs with NES ≥ 3 were selected and ranked. NES, AUC and candidate TFs of the top 60 motifs were shown.
